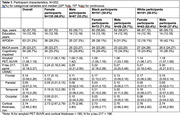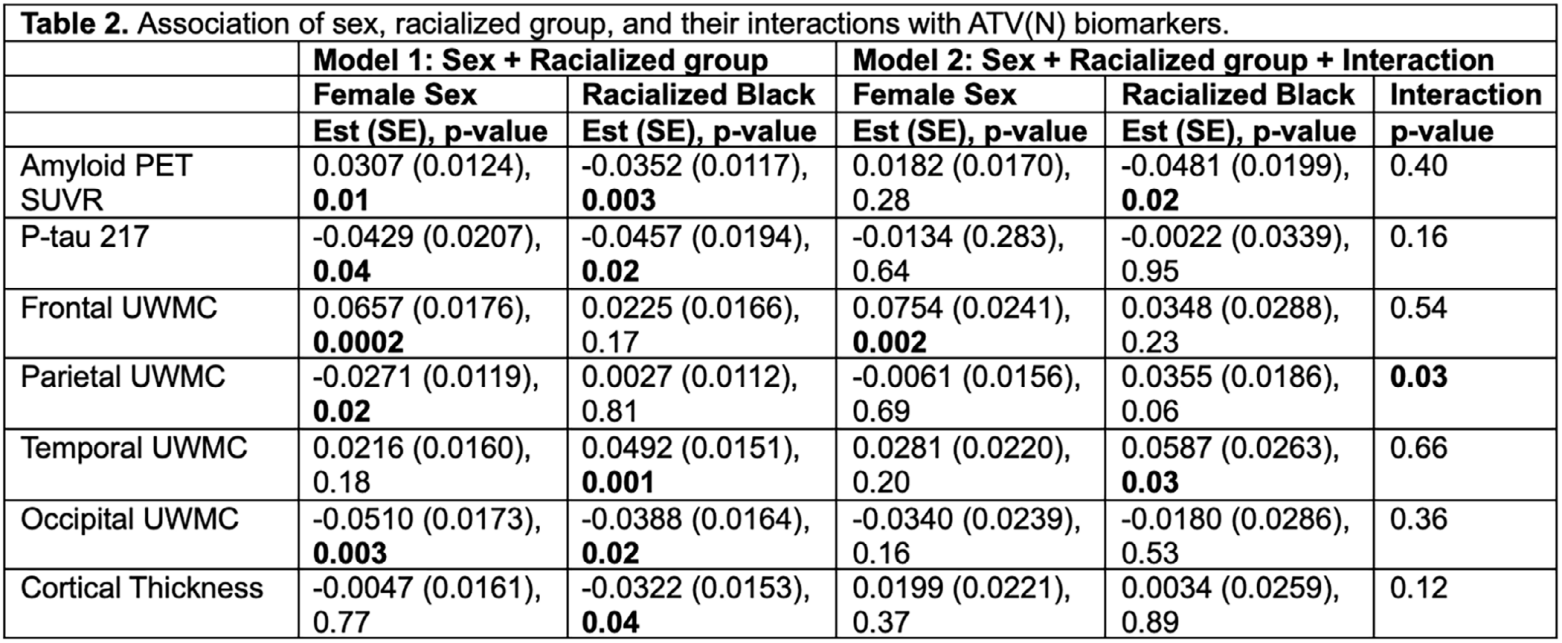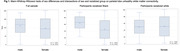# Intersections of sex and racialization on ATV(N) biomarkers

**DOI:** 10.1002/alz.087089

**Published:** 2025-01-09

**Authors:** C. Elizabeth Shaaban, Alexandria C. Reese, Sarah K. Royse, Beth E. Snitz, Thomas K Karikari, James Hengenius, Theodore Huppert, Rebecca E. Roush, Geraldine Cisneros, Katey Potopenko, James T Becker, Annie Cohen

**Affiliations:** ^1^ University of Pittsburgh, Pittsburgh, PA USA; ^2^ University of Pittsburgh Alzheimer's Disease Research Center (ADRC), Pittsburgh, PA USA; ^3^ School of Medicine, University of Pittsburgh, Pittsburgh, PA USA; ^4^ Department of Psychiatry, School of Medicine, University of Pittsburgh, Pittsburgh, PA USA; ^5^ CHU Sainte‐Justine Research Centre, Université de Montréal, Montréal, QC Canada; ^6^ University of Pittsburgh School of Medicine, Pittsburgh, PA USA

## Abstract

**Background:**

Differences in Alzheimer’s disease (AD) amyloid, tau, vascular, and neurodegeneration (ATV(N)) biomarkers by sex and racialized group have been reported, but little is known about the intersections of sex and racialization on these biomarkers.

**Methods:**

Participants in this cross‐sectional analysis are from the Connectomics of Brain Aging study, a Human Connectome Project protocol evaluating brain structure and function in aging and AD. We measured A using global amyloid SUVR from ^11^C‐PiB PET, T using plasma p‐tau 217, V using lobar unhealthy white matter connectivity (UWMC), a novel measure indicating the proportion of WM connections impacted by white matter hyperintensities (WMH), and N using cortical thickness from an AD‐based meta‐region of interest. We tested for intersectional effects of sex and racialized group on ATV(N) biomarkers in robust regressions by testing models with and without sex*racialized group interaction terms.

**Results:**

The sample included N=202 participants with the following characteristics: age, 62 years; female sex, 67%; Black racialization, 50%; education, 14 years; APOE4+, 31%; cognitively impaired, 27%; Table 1. In robust regression models we found that women had significantly more amyloid and frontal UWMC, and less tau and parietal and occipital UWMC than men; participants racialized as Black had significantly more temporal lobe UWMC and significantly less occipital lobe UWMC, amyloid, tau, and cortical thickness than participants racialized as white (Table 2). Intersectional effects were found only for parietal lobe UWMC (p for interaction=0.03; Table 2), such that men racialized as Black had the greatest parietal UWMC, while men and women racialized as white did not vary in their parietal UWMC (Figure 1). Results with lobar WMH instead of UWMC were similar.

**Conclusion:**

Our results confirm ATV(N) biomarker differences by sex and racialized group. They suggest that men racialized as Black may be uniquely at risk for UWMC in the parietal lobe but found no intersectional effects on other biomarkers. Future work will investigate specific WM tracts involved in the parietal UWMC and associated risk factors such as structural and social determinants of health as well as cardiovascular risk factors and comorbidities.